# The Optimal Design of Modulation Angular Rate for MEMS-Based Rotary Semi-SINS

**DOI:** 10.3390/mi10020111

**Published:** 2019-02-10

**Authors:** Jiayu Zhang, Jie Li, Xiaorui Che, Xi Zhang, Chenjun Hu, Kaiqiang Feng, Tingjin Xu

**Affiliations:** 1 National Key Laboratory for Electronic Measurement Technology, North University of China, Taiyuan 030051, China; jyz1606108@gmail.com (J.Z.); zhangxi@nuc.edu.cn (X.Z.); b1506011@st.nuc.edu.cn (K.F.); s1606023@st.nuc.edu.cn (T.X.); 2 Avic Shaanxi Huayan Aero-Instrument Co., Ltd., Hanzhong 723102, China; xiaoruiche@gmail.com; 3 Suzhou Fashion Nano Technology Co., Ltd., Suzhou 215000, China; hcj74839@gmail.com

**Keywords:** rotation semi-SINS, rotation modulation, MEMS, rotation speed error

## Abstract

In previous studies, the semi-strapdown inertial navigation system (SSINS), based on microelectromechanical system (MEMS) sensors, had realized cross-range measurement of attitude information of high-spinning projectiles through construction of a “spin reduction” platform of the roll axis. However, further improvement of its measurement accuracy has been difficult, due to the inertial sensor error. In order to enhance the navigational accuracy, a periodically rotating method is utilized to compensate for sensor error, which is called rotation modulation. At present, the rotation scheme, as one of the core technologies, has been studied by a lot of researchers. It is known that the modulation angular rate is the main factor affecting the effectiveness of error modulation. Different from the long-endurance and low-dynamic motion characteristics of ships, however, the short-endurance and high-dynamic characteristics of the high-spinning projectile not only require the modulation angular rate to be as fast as possible but, also, the influence of the rotation speed error caused by rotating mechanism errors cannot be ignored. Combined with the rotation speed error of the rotating mechanism, this paper explored the relationship between modulation angular rate, device error, and the navigation error, and then proposed a design method for optimal modulation angular rate. Experiments were carried out to validate the performance of the method. In addition, the proposed method is applicable for rotation modulation systems with different types of motors as the rotating mechanism.

## 1. Introduction

With the increasing importance of conventional ammunition guidance in modern war, developing technology to realize the navigation control and trajectory real-time correction of conventional ammunition is key [1]. Accurate measurement of attitude information of the guided projectile is the basis for precise guidance and control. The inertial navigation system (INS) is a fully autonomous navigation system based on Newton’s law of inertia, and can provide attitude, velocity, and position information without the influence of terrain, weather, and time [2], therefore, INS plays an irreplaceable role in conventional weapon guidance. At present, microelectromechanical system (MEMS) sensors are suitable for projectile-borne applications in terms of size, cost, consumption, and anti-overload ability [3]. When a MEMS strapdown inertial measurement system is used to measure the attitudes of high-spinning projectiles, the large dynamic range of MEMS inertial sensor is required, meanwhile, the high precision of MEMS inertial sensors is very important [4]. However, the fact that the noise properties of a typical MEMS gyroscope lead to large accumulative errors makes it difficult for the strapdown INS (SINS) to accurately measure the roll angle. Due to many factors, such as the manufacturing level of MEMS inertial devices, integrated design of high-performance detection circuits, and high-reliability packaging, improving the accuracy of MEMS sensors in a short time is difficult [5].

In practice, due to factors such as the application environment and sensor error, the system usually exhibits nonlinear and non-Gaussian problems, and the common filtering method is no longer applicable. Instead, an advanced filtering algorithm plays the central role. Gustafsson et al. explored the relationship between extended Kalman filter (EKF) and unscented Kalman filter (UKF). For nonlinear filtering problems where the nonlinearity is severe compared to the prior state information, the performance of UKF is significantly better than EKF [6]. Another good way to deal with nonlinear filtering problems is particle filter (PF), which is a sequential Monte Carlo methodology where the basic idea is the recursive computation of relevant probability distributions using the concepts of importance sampling and approximation of probability distributions with discrete random measures, and good anti-noise and stability [7]. Martino et al. designed a sequential Monte Carlo scheme for the dual purpose of Bayesian inference and model selection, and used interacting parallel particle filters, each one addressing a different model. The robustness is greatly improved [8].

At the same time, the high accuracy measurement of high-spinning projectile attitude has attracted a lot of attention in recent years. Schuler et al. proposed the method of measuring the rotational motion of the carrier with the accelerometers in 1967, which was also known as gyro-free SINS (GF-SINS) [9]. In theory, GF-SINS requires a minimum of 6 sensors. However, it has been shown in various works of literature that at least nine accelerometers, or even twelve, are required to improve the stability and accuracy of the system. Moreover, due to the precession and nutation of the projectile during high-dynamic flight, its normal acceleration will be affected by the aerodynamic lift, which affects the accuracy of the speed measurement.

With the development of control and guided technology, many new foreign weapons adopt the GPS/INS in recent years. Fairfax et al. proposed an algorithm for estimating the position and velocity of projectiles, which established the point mass (PM) flight dynamics model in the EKF model using the inertial measurement unit (IMU), attitude, and global positioning system (GPS) information. Measurement parameters are estimated in flight with a moving average filter (MAF) using GPS/INS loose coupling [10]. However, the method of GPS/INS integrated navigation still relies on external information and cannot achieve complete autonomous navigation. Similarly, the solar azimuth measurement method can also be used to measure the attitude of the projectile, and has the ability to resist high overload, but it is susceptible to the weather.

**Figure 1:**
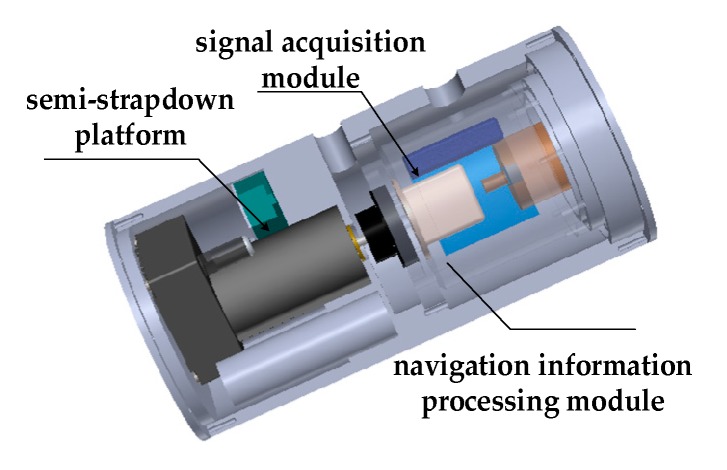
The structure of strapdown inertial navigation system (SINS).

Above all, we still choose the MEMS-IMU (MIMU) to measure the velocity, position, and attitude information. To solve the contradiction between high range and high precision, the concept of semi-strapdown inertial navigation system (SSINS) was proposed by the Key Laboratory of Instrumentation Science and Dynamic Measurement [11]. The structure of the system is shown as Figure 1, which is mainly composed of semi-strapdown platform, signal acquisition module, and navigation information processing module.

In contrast to SINS, the signal acquisition module is not rigidly attached to the carrier but, rather, is connected to the carrier via the servo motor. The main function of the semi-strapdown platform is to realize roll isolation between MEMS-IMU(MIMU) and carrier, which makes the MIMU work in the low-dynamic environment, so that the gyroscope with smaller range and higher accuracy can be utilized to measure rolling angular rate. However, SINS is still prone to sensor error accumulation with time, resulting in deterioration of the navigation solution [12]. Fortunately, the rotation modulation technique can serve as an alternative way to reduce navigation errors, which uses its own rotating mechanism to rotate in a certain order, and the constant bias of the inertial devices are modulated into the zero mean form [13,14,15]. Therefore, on the basis of reducing rotation in SSINS, the motor drives the MIMU to rotate at the appropriate angular rate to complete the single-axis rotation modulation, called rotary semi-strapdown inertial navigation system (RSSINS).

Reference [16] shows that the faster the modulation angular rate is, the better the constant error suppression effect is. Nevertheless, the symmetry scale factor error and installation error of the gyroscope can not only be modulated, but also generate instantaneous velocity errors related to the modulation angular rate [17,18]. For MEMS-based rotary inertial navigation system, due to the low accuracy of MEMS gyros, a faster modulation angular rate is required for error suppression [19]. However, in practical applications, the faster rotation speed will introduce rotation speed errors in the rotating mechanism, resulting in the modulation effect being destroyed [20]. As a result, the design of the rotation modulation angular rate has become a hotspot of rotation modulation. Che deduced the influence of IMU motion change process on navigation precision in detail, and analyzed the error characteristics of the forward and reverse rotation scheme, but did not propose a method for designing the optimal modulation angular rate [21]. Combined with the error characteristics of fiber optic gyro, Yu proposed a method based on Laplace transform and its inverse transform to design the optimal angular rate [22].

However, due to the application characteristics of short-endurance and semi-strapdown, the rotating mechanism is required to follow the high-dynamic changes of the projectile in real-time, which inevitably generates rotating mechanism errors, thus destroying the modulation effect and even causing greater errors. Until now, have been few studies on the optimal design of modulation angular rate for MEMS-based rotation inertial navigation system (RINS). Therefore, the setting of the modulation angular rate as one of the key technologies for the rotation modulation scheme cannot be avoided. In this article, combined with factors such as the measurement environment, motor performance, carrier movement, device error characteristic, and others, a novel modulation angular rate design method was proposed.

In this study, combined with the characteristics of MEMS sensor and rotation speed error of rotating mechanism, we propose a design method for optimal modulation angular rate suitable for missile-borne navigation systems. The rest of this paper is organized as follows. Section 2 introduces the system working principle and systematic error model. Combined with the measurement environment, motor performance, and device error characteristic, the method to obtain optimal modulation angular rate is presented in Section 3. The optimal modulation angular rate is obtained, and the tests are given to verify error suppression performance in Section 4. Section 5 is the conclusion.

## 2. System Working Principle

### 2.1. The Principle behind the Rotary Semi-Strapdown Inertial Navigation System (RSSINS)

Differently from SINS, a gyro with wide range is mounted on the roll axis to measure carrier spin rate in RSSINS and, then, the information measured by it is processed and converted into a control signal to drive the motor to rotate in an opposite direction to the carrier, so as to provide a low-dynamic environment for the MIMU.

**Figure 2:**
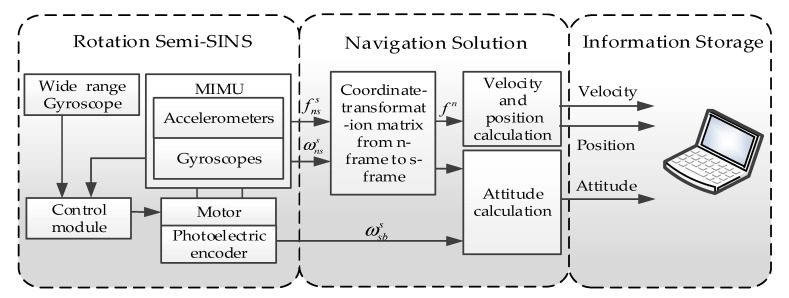
The principle block diagram of rotary semi-strapdown inertial navigation system (RSSINS).

On the basis of “spin reduction”, due to the low precision of the wide-range gyro, the MIMU installed on the signal acquisition module can be sensitive to the residual angular rate, and it is fed back to the control module for adjusting the motor output in real-time, to drive the MIMU to complete the rotation modulation. At the same time, the photoelectric encoder is used to measure the relative rotation angle between the carrier and the MIMU, thus realizing the solution of carrier navigation parameters. The principle behind the RSSINS is shown in Figure 2. In this case, isolation of the MIMU and the high-spinning of the carrier’s roll axis is implemented by the mechanical structure and control method.

In general, the mechanization algorithm of RSSINS is similar to the one of conventional SINS. Gyro and accelerometer are used to measure the angular motion information and line motion information of the carrier, respectively, and then calculate the attitude, speed, and position information. Differently from the solution algorithm of SINS, the measurement information of the encoder shall be introduced into the navigation solution loop. Since there is no relative displacement between the IMU and the carrier, the MIMU’s position is the carrier’s position. Therefore, the angular rate and specific force measured by the MIMU are directly introduced into the solution algorithm, such that the attitude matrix between the MIMU coordinate system and navigation coordinate system is obtained, from which the attitude of MIMU can be obtained. Then, the carrier attitude needs to be calculated according to the relative rotation of the carrier and the MIMU measured by the encoder. In this navigation solution loop, the measurement error of the relative rotation angle will result in roll angle error, but the relative rotation angle error will not be introduced into the solution loop and have no effect on the velocity and position accuracy [23].

### 2.2. Error Model

In order to analyze the error propagation characteristic of the RSSINS, the frames are defined as follows.

(1) s-frame: MIMU frame, with X—Forward, Y—Up, and Z—Right pointing orientations;

(2) b-frame: carrier’s body frame, with X—Roll, Y—Yaw, and Z—Pitch pointing orientations;

(3) n-frame: navigation reference frame, with N–U–E (North–Up–East) pointing orientations;

(4) b′-frame: for the convenience of description, in addition, $b^{\prime }$-frame is defined as a virtual coordinate system, which refers to the s-frame before rotation modulation is introduced.

For MIMU, the influence of the variation of the gravitational field and the curvature radius of the Earth’s surface on the inertial navigation system error is negligible when the carrier is working for a short time [24]. Since the sensors selected by this system cannot sense the Earth’s rotation speed, the cross-coupling term generated by it can be ignored. Consequently, the error propagation equations of RSSINS have been derived as follows.

(1) $$\delta {\dot{V}}^{n}=-\phi^{n}\times f^{n}+C_{b}^{n}C_{b^{\prime }}^{b}C_{s}^{b^{\prime }}([\delta K_{A}+\delta G]f^{s}+\nabla^{s})$$

(2) $$\dot{\phi }=-C_{b}^{n}C_{b^{\prime }}^{b}C_{s}^{b^{\prime }}((\delta K_{G}+\delta G)\times \omega_{ns}^{s}+\varepsilon^{s})$$

(3) $$\delta \dot{L}=\frac{\delta V_{N}}{R_{M}+h}-\delta h\frac{V_{N}}{{\left(R_{M}+h\right)}^{2}}$$

(4) $$\delta \dot{\lambda }=\frac{\delta V_{E}}{R_{N}+h}\sec L+\delta L\frac{V_{E}}{R_{N}+h}\tan L\sec L-\delta h\frac{V_{E}\sec L}{{\left(R_{N}+h\right)}^{2}}$$

(5) $$\delta \dot{h}=\delta V_{U}$$

where

$$\begin{array}{l} \delta {\dot{V}}^{n}=\left(\begin{array}{c} \delta {\dot{V}}_{N}\\ \delta {\dot{V}}_{U}\\ \delta {\dot{V}}_{E} \end{array}\right);V^{n}=\left(\begin{array}{c} V_{N}\\ V_{U}\\ V_{E} \end{array}\right);f^{n}=\left(\begin{array}{c} f_{N}\\ f_{U}\\ f_{E} \end{array}\right);f^{s}=\left(\begin{array}{c} f_{x}\\ f_{y}\\ f_{z} \end{array}\right);\phi =\left(\begin{array}{c} \phi_{N}\\ \phi_{U}\\ \phi_{E} \end{array}\right);\delta K_{A}=\left(\begin{array}{ccc} \delta K_{Ax} & 0 & 0\\ 0 & \delta K_{Ay} & 0\\ 0 & 0 & \delta K_{Az} \end{array}\right);\delta K_{G}=\left(\begin{array}{ccc} \delta K_{gx} & 0 & 0\\ 0 & \delta K_{gy} & 0\\ 0 & 0 & \delta K_{gz} \end{array}\right);\\ \delta A=\left(\begin{array}{ccc} 0 & \delta K_{Axy} & \delta K_{Axz}\\ \delta K_{Ayx} & 0 & \delta K_{Ayz}\\ \delta K_{Azx} & \delta K_{Azy} & 0 \end{array}\right);\delta G=\left(\begin{array}{ccc} 0 & \delta K_{gxy} & \delta K_{gxz}\\ \delta K_{gyx} & 0 & \delta K_{gyz}\\ \delta K_{gzx} & \delta K_{gzy} & 0 \end{array}\right);\phi^{n}=\left[\begin{array}{ccc} 0 & -\phi_{E} & \phi_{U}\\ \phi_{E} & 0 & -\phi_{N}\\ -\phi_{U} & \phi_{N} & 0 \end{array}\right];\begin{array}{c} \nabla^{s}={\left(\begin{array}{ccc} \nabla_{x}^{s} & \nabla_{y}^{s} & \nabla_{z}^{s} \end{array}\right)}^{T};\\ \varepsilon^{s}={\left(\begin{array}{ccc} \varepsilon_{x}^{s} & \varepsilon_{y}^{s} & \varepsilon_{z}^{s} \end{array}\right)}^{T}; \end{array} \end{array}$$

$\delta \dot{V}$ represents the carrier velocity error in n-frame; $V^{n}$ represents the carrier velocity in n-frame; $\phi_{E}$, $\phi_{N}$, and $\phi_{U}$ represent the attitude errors between n-frame and mathematical platform frame; $f^{n}$ and $f^{s}$ represent the carrier’s specific force in n-frame and s-frame, respectively; $\delta K_{A}$ and δA represent the scale factor error and the installation error of accelerometers; $\delta K_{G}$ and δG represent the scale factor error and the installation error of gyroscopes; $\nabla^{s}$ and $\varepsilon^{s}$ represent the constant bias of accelerometers and gyroscopes, respectively; $C_{A}^{B}$ is the transformation matrix from A-frame to B-frame; δL, δλ, and δh represent the error of the local latitude, longitude, and height; and *L* represents the local latitude.

## 3. Design of Optimal Modulation Angular Rate

### 3.1. System Error Analysis

Unlike the conventional rotary SINS, the servo motor produces a large speed to isolate the high-spinning of carrier’s roll axis in RSSINS, which inevitably generates the rotation speed error. Therefore, for the optimal design of modulation angular rate, in addition to the constant bias, scale factor error, and installation error, the rotation speed error should also be considered. This section will investigate the relationship between the modulation angular rate and system performance.

Based on the error model mentioned in Section 2.2, the errors model of RSSINS are shown in Equation (6):

(6) $$\dot{X}(t)=AX(t)+W(t),$$

where

$$\begin{array}{c} X(t)={\left(\begin{array}{ccccccccc} \phi_{N} & \phi_{U} & \phi_{E} & \delta V_{N} & \delta V_{U} & \delta V_{E} & \delta L & \delta \lambda & \delta h \end{array}\right)}^{T};\\ A=\left[\begin{array}{ccc} 0_{3\times 3} & 0_{3\times 3} & 0_{3\times 3}\\ A_{21} & 0_{3\times 3} & 0_{3\times 3}\\ 0_{3\times 3} & A_{32} & 0_{3\times 3} \end{array}\right];A_{21}=\left[\begin{array}{ccc} 0 & 0 & g\\ 0 & 0 & 0\\ -g & 0 & 0 \end{array}\right];A_{32}=\left[\begin{array}{ccc} \frac{1}{R_{M}} & 0 & 0\\ 0 & 0 & \frac{\sec(L)}{R_{N}}\\ 0 & 1 & 0 \end{array}\right]\\ W(t)=\left[\begin{array}{ccccccccc} W(1) & W(2) & W(3) & W(4) & W(5) & W(6) & W(7) & W(8) & W(9) \end{array}\right]\\ W(1)=-\varepsilon_{x}^{s}+\delta K_{gx}\omega ;\\ W(2)=-\varepsilon_{z}^{s}\sin(\omega t)-\varepsilon_{y}^{s}\cos(\omega t)+\delta K_{gyx}\omega \cos(\omega t)+\delta K_{gzx}\omega \sin(\omega t);\\ W(3)=\varepsilon_{y}^{s}\sin(\omega t)-\varepsilon_{z}^{s}\cos(\omega t)+\delta K_{gzx}\omega \cos(\omega t)-\delta K_{gyx}\omega \sin(\omega t);\\ W(4)=\nabla_{x}^{s}+\delta K_{Axy}g\cos\omega t+\delta K_{Axz}g\sin\omega t;\\ W(5)=\nabla_{y}^{s}\cos\omega t+\nabla_{z}^{s}\sin\omega t+\delta K_{Ay}g\cos^{2}\omega t+\delta K_{Ayz}g\sin\omega t\cos\omega t+\delta K_{Azy}g\cos\omega t\sin\omega t+\delta K_{Az}g\sin^{2}\omega t;\\ W(6)=-\nabla_{y}^{s}\sin\omega t+\nabla_{z}^{s}\cos\omega t-\delta K_{Ay}g\cos\omega t\sin\omega t-\delta K_{Ayz}g\sin^{2}\omega t+\delta K_{Azy}g\cos^{2}\omega t+\delta K_{Az}g\sin\omega t\cos\omega t;\\ W(7)=0;W(8)=0;W(9)=0; \end{array}$$

Ignoring the initial alignment error, and solving the inhomogeneous linear differential equations with the aid of Mathematica, the results are as follows.

(7) $$\phi E(t)=\frac{-(\varepsilon_{y}^{s}-K_{gyx}\omega )(-1+\cos(t\omega ))+(-\varepsilon_{z}^{s}+K_{gzx}\omega )\sin(t\omega )}{\omega };$$

(8) $$\phi N(t)=t(K_{gx}\omega -\varepsilon_{x}^{s});$$

(9) $$\phi U(t)=\frac{\left(K_{gyx}\omega -\varepsilon_{y}^{s})\sin(t\omega )+(\varepsilon_{z}^{s}-K_{gzx}\omega )(\cos(t\omega )-1\right)}{\omega };$$

(10) $$\delta VE(t)=\frac{1}{4\omega }(4\nabla_{y}^{s}\cos(t\omega )-4\nabla_{y}^{s}+4\nabla_{z}^{s}\sin(t\omega )+g(K_{Ay}-K_{Az})\cos(2t\omega )+g(-K_{Ay}+2t\omega (-K_{Ayz}+K_{Azy}+K_{gx}t\omega -\varepsilon_{x}^{s}t)+K_{Az})+g(K_{Ayz}+K_{Azy})\sin(2t\omega ));$$

(11) $$\delta VN(t)=\frac{1}{\omega^{2}}((\nabla_{x}^{s}-gK_{gyx})t\omega^{2}-\varepsilon_{z}^{s}g+g\omega (K_{Axz}+K_{gzx}+\varepsilon_{y}^{s}t)+g\cos(t\omega )(\varepsilon_{z}^{s}-\omega (K_{Axz}+K_{gzx}))+g\sin(\omega t)(\varepsilon_{y}^{s}-(K_{gyx}+K_{Axy})\omega ));$$

(12) $$\delta VU(t)=\frac{1}{4\omega }(4\nabla_{y}^{s}\sin(t\omega )-4\nabla_{z}^{s}\cos(t\omega )+4\nabla_{z}^{s}+g2t\omega (K_{Ay}+K_{Az})+gKA_{yz}+gK_{Azy}+g(K_{Ay}-K_{Az})\sin(2t\omega )-g(K_{Ayz}+K_{Azy})\cos(2t\omega ));$$

(13) $$\begin{array}{l} \delta L(t)=\frac{1}{2RM\omega^{3}}(-2\varepsilon_{y}^{s}g+2g(K_{Axy}+K_{gyx}-\varepsilon_{z}^{s}t)\omega +gt(2(K_{Axz}+K_{gzx})+\varepsilon_{y}^{s}t)\omega^{2}+(\nabla_{x}^{s}-gK_{gyx})t^{2}\omega^{3}+2g(\varepsilon_{y}^{s}-(K_{Axy}+K_{gyx})\omega )cos(t\omega )\\ +2g(\varepsilon_{z}^{s}-(K_{Axz}+K_{gzx})\omega )sin(t\omega )); \end{array}$$

(14) $$\begin{array}{l} \delta \lambda (t)=\frac{1}{24R_{N}\omega^{2}}\sec(L)(-6t\omega (4\nabla_{y}^{s}+gK_{Ay}-gK_{Az})+24\nabla_{y}^{s}\sin(t\omega )-24\nabla_{z}^{s}\cos(t\omega )+24\nabla_{z}^{s}+3g(K_{Ay}-K_{Az})\sin(2t\omega )\\ -2gt^{2}\omega^{2}(3K_{Ayz}-3K_{Azy}+2\varepsilon_{x}^{s}t)-3g(K_{Ayz}+K_{Azy})\cos(2t\omega )+3g(K_{Ayz}+K_{Azy})+4gK_{gx}t^{3}\omega^{3}); \end{array}$$

(15) $$\begin{array}{l} \delta h(t)=\frac{1}{8\omega^{2}}(-8\nabla_{y}^{s}\cos(t\omega )+8\nabla_{y}^{s}+8\nabla_{z}^{s}t\omega -8\nabla_{z}^{s}\sin(t\omega )+g(2t^{2}\omega^{2}(K_{Ay}+K_{Az})+K_{Ay}+2t\omega (K_{Ayz}+K_{Azy})-K_{Az})\\ +g(K_{Az}-K_{Ay})\cos(2t\omega )-g(K_{Ayz}+K_{Azy})\sin(2t\omega )); \end{array}$$

**Figure 3:**
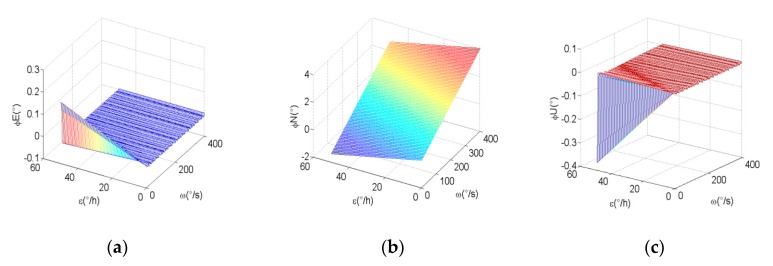

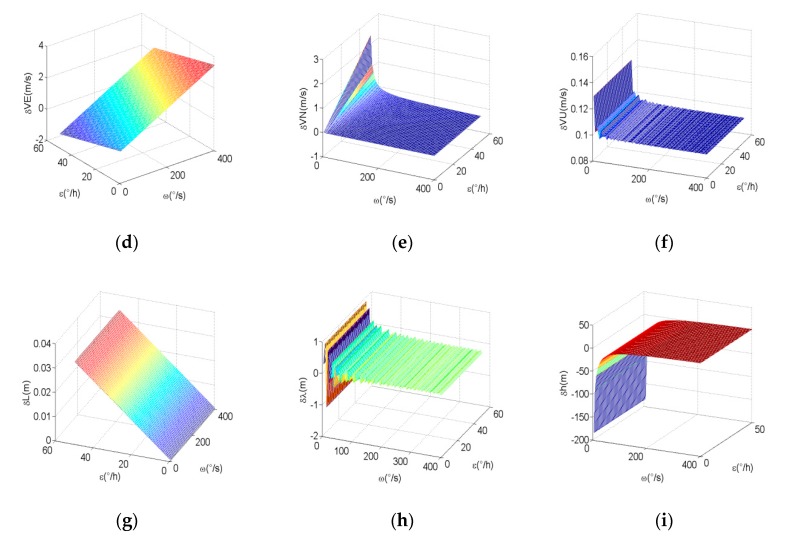
(**a**–**c**) The relationship curves of east, north, and up attitude errors with the constant bias of gyro and modulation angular rate, respectively. (**d**–**f**) The relationship curves of east, north, and up velocity errors with the constant bias of gyro and modulation angular rate, respectively. (**g**–**i**) The relationship curves of latitude, longitude and height errors with the constant bias of gyro and modulation angular rate, respectively.

It can be seen from Equations (7)–(15) that the navigation error is related to modulation angular rate and inertial sensor errors. The scale factor error and installation error of MIMU can be accurately calibrated and compensated, therefore, constant bias is a major factor in the decreasing navigation precision. To more intuitively express the relationship between system error, modulation angular rate, and constant bias, we assume a scale factor error of 200 ppm, installation error of 20 arcmin, and accelerometer bias of 0.1 mg. The relationship curves of the navigation errors, constant bias of the gyroscope, and modulation angular rate are shown in Figure 3.

From Figure 3, we can see that modulation angular rate and the constant bias of gyro have an obvious effect on north attitude error, east velocity error, and latitude error. Therefore, the analysis of optimal modulation angular rate mainly focuses on these errors. Known from Equations (8), (10), and (13), these errors can be expressed as a function of constant bias, scale factor, installation error, and the modulation angular rate. For the purpose of ensuring the minimum of navigation errors, the modulation angular rate should match the errors of constant bias, scale factor error, and installation error.

### 3.2. Motor Error Analysis

For realizing the dual function of “spin reduction” and “rotation modulation” in the high-spinning condition, the motor is required to follow the high-dynamic changes of the projectile in real-time, so that the angular rate of the motor outputs cannot be so good as in the theoretical analysis, which inevitably affects the error modulation effect. It can be seen from the analysis of rotation speed error of rotating mechanism in [25] that the gyro drift perpendicular to the rotation axis cannot be completely offset within a modulation period, and generates attitude errors which accumulate over time.

Due to the function of spin reduction, the modulation angular rate error—caused by the motor speed error operating in high-speed motion—seriously affects the modulation effect. In order to analyze the influence of modulation angular rate error on navigation accuracy, it is necessary to establish the relationship between modulation angular rate and corresponding rotation speed error. To simplify the analysis, assume that the speed of the projectile is 15 r/s. By presetting different modulation angular rates to the system, the motor drives the information acquisition module of the system to rotate at different speeds to obtain the motor speed error. Through many experiments, the actual error of the motor can be obtained and shown in Table 1, thus establishing the relationship between the modulation angular rate and the rotation speed error.

It can be seen from Table 1 that the rotation speed error gradually increases with rotation speed. The relationship between the modulation angular rate and the rotation speed error can be expressed as Equation (16) by conics fitting.

(16) δω=0.02796×ω+8.513

Therefore, the optimal modulation angular rate is analyzed and determined in combination with motor speed error, actual device error, system installation error, and others. According to the application background of RSSINS and MEMS device characteristics, the relevant parameters in the system are as follows. In the projectile-borne conditions, the carrier has a shorter flight time, where t = 40 s is taken. In the actual RSSINS, the constant bias of gyros is 24°/h, the constant bias of accelerometers is 0.1 mg, the scale factor error of accelerometers and gyros are 200 ppm, the installation error of accelerometers and gyros are 30 arcmin, the local latitude L is 38°, and the gravity magnitude g is 9.7997 m/s^2^. Therefore, we can obtain the relationship curve of north attitude error, east velocity error, latitude error, and the modulation angular rate, respectively.

**Figure 4:**
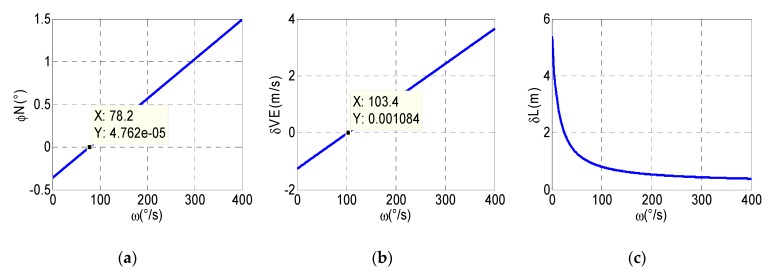
(**a**) The relationship curve of north attitude error and modulation angular rate. (**b**) The relationship curve of east velocity error and modulation angular rate. (**c**) The relationship curve of latitude error and modulation angular rate.

As can be seen from Equation (8), the north attitude error is not only related to the constant bias of gyro but also related to the coupling of scale factor error and modulation angular rate. Therefore, as shown in Figure 4, the absolute value of the north attitude error does not vary monotonously but, rather, tends to initially decrease and then increase with increasing modulation angular rate, and when the modulation angular rate is 78.2°/s, the north attitude error is minimized. Similarly, east velocity error is greatly affected by the modulation angular rate, and when the modulation angular rate is 103.4°/s, the error suppression works best. In the meantime, it can be seen that the latitude error decreases as the modulation angular rate increases. Hence, after all the consideration, 103°/s is selected as the optimal modulation angular rate for the RSSINS.

## 4. Test and Results Analysis

The optimal design method of the modulation angular rate is proposed in Section 3. The theoretical optimal modulation angular rate is obtained in terms of this method. In order to verify the correctness of the former conclusions, experiments are implemented by using a high-precision three-axis flight simulator, the technical parameters of which are shown in Table 2.

**Figure 5:**
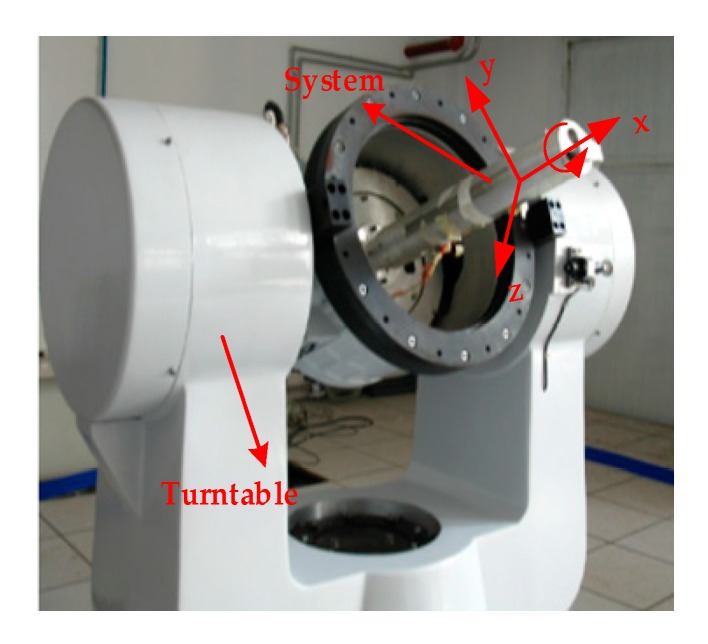
RSSINS installation on three-axis flight simulator.

The RSSINS is installed on the high-precision three-axis flight simulator as shown in Figure 5, which is used to simulate the carrier’s attitude change. With the sensitive axes of MIMU defined as the *x*-axis pointing forward, the *z*-axis pointing right, and the *y*-axis pointing up, the rotation of the inner frame rotates the system about its *x*-axis.

At the same time, the characteristics of the MIMU in the system is shown in Table 3.

In Section 3.2, the average value of motor rotation speed error is used to analyze the impact on modulation effect. In order to more accurately obtain the optimal modulation angular rate, in addition to the theoretical optimal modulation angular rate of 103°/s, the angular rates of 101°/s, 102°/s, 104°/s, and 105°/s were also selected as the modulation angular rate for testing, and compared with the testing results. Furthermore, two groups were added to the test, with 60°/s and 120°/s as the modulation angular rates, respectively, to analyze the error suppression effect.

**Figure 6:**
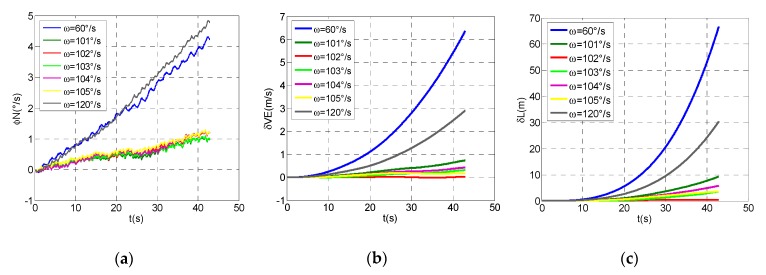
(**a**) The north attitude errors at different modulation angular rates. (**b**) The east velocity errors at different modulation angular rates. (**c**) The latitude errors at different modulation angular rates.

In all the experiments, the inner frame of three-axis flight simulator simulates the carrier rolling motion with the speed of 5400°/s; the mid-frame simulates the carrier’s pitching motion, with the range between +45° and −45°; the modulation time is 40 s, and the reciprocating rotation scheme is employed. Comparing the navigation information measured by RSSINS with the standard information provided by the three-axis flight simulator, the navigation parameter measurement errors of the system at different modulation angular rates can be obtained to analyze the error suppression performance. Though the analysis of experimental results, the errors of north attitude, east velocity, and latitude at different modulation angular rates are shown in Figure 6, and the maximum values of each error are shown in Table 4.

Compared with the modulation angular rate of 60°/s and 120°/s, we can observe that the error suppression effect is better when the modulation angular rates are between 101°/s and 105°/s, but it is not the best, which proves the validity of the analysis in Section 3.2, that is, 102°/s is not the best angular rate of the attitude error suppression effect. Meanwhile, the modulation angular rate error results in the constant bias of gyro not being completely eliminated, so that the residual error accumulates with time, which is one of the reasons for the divergence of the attitude error. In addition, the attitude accuracy can be improved by compensating the rotation speed error. Given the limited space available, it will not be described in detail here. Similarly, through analyzing the characteristics of east velocity error and latitude error, the navigation performance is optimal when the modulation angular rate is 102°/s, which is different from the modulation angular rate obtained by the method mentioned in the Section 3. The difference between them is mainly caused by the accuracy of the motor rotation speed error model, whereas the accurate modeling of rotation speed error makes it more difficult to analyze and calculate. Hence, the method proposed in this paper can be used to determine the optimal angular rate simply by theoretical calculation and experimental verification, and it is suitable for rotary navigation systems with different types of motors as the rotating mechanism.

## 5. Conclusions

In this paper, an optimal design method for the modulation angular rate is proposed for high-dynamic and short-endurance cases. First of all, the concept and working principle of RSSINS were introduced, and the systemic error equations were developed. Secondly, combined with sensor errors and motor rotation speed error model, the optimal modulation angular rate was determined based on the relationship between the system errors and the modulation angular rate. In order to verify the validity and correctness of the optimal angular rate, a series of comparative experiments were carried out, and the results show that the optimal angular rate obtained by the proposed method had good error suppression performance. However, we simply model the motor rotation speed error, rather than exploring an accurate motor rotation speed error model. If the motor rotation speed error is accurately modeled, it needs to be analyzed individually for different types of motors, which undoubtedly increases the workload and computational complexity. Hence, the modulation angular rate can be theoretically determined, roughly, by the method proposed in this paper, and the optimal modulation angular rate can be obtained through experimental verification.

## Figures and Tables

**Table 1:** Theoretical and practical rotation angular rate data.

Modulation Angular Rate (°/s)	Theoretical Rotation Speed (°/s)	Actual Rotation Speed (°/s)	Rotation Speed Error (°/s)
0	5400	5390.2898	9.7102
50	5450	5440.1452	9.8548
100	5500	5489.9626	10.6374
150	5550	5538.2572	11.8428
200	5600	5586.0580	13.9420
250	5650	5634.7351	15.2649
300	5700	5683.0571	16.9426
350	5750	5731.2597	18.7403
400	5800	5779.9926	20.0074

**Table 2:** Technical parameters of tri-axial flight simulator.

Position Accuracy (°)	Rotation Rate Accuracy (°/s)	Rotation Rate (°/s)		
		Inner Frame	Middle Frame	Outer Frame
0.001	0.001	0.001–12000	0.001–400	0.001–400

**Table 3:** Characteristics of MEMS-IMU (MIMU).

Characteristics	Range	Bias	Random Walk
Gyroscope (*x* axis)	±200°/s	24°/h	0.28 ${}^{\circ}/\sqrt{h}$
Gyroscopes (*y*, *z* axis)	±75°/s	24°/h	0.28 ${}^{\circ}/\sqrt{h}$
Accelerometer (*x* axis)	±200 g	5 mg	150 ug$\sqrt{Hz}$
Accelerometer (*y*, *z* axis)	±10 g	1 mg	90 ug/$\sqrt{Hz}$

**Table 4:** Test data.

Rotation Angular Rate (°/s)	North Attitude Error (°)	East Velocity Error (m/s)	Latitude Error (m)
60	4.229	6.359	66.63
101	0.9994	0.7356	9.292
102	1.193	0.0802	1.4714
103	1.01	0.3126	3.589
104	1.202	0.4296	5.83
105	1.21	0.1782	3.709
120	4.79	2.895	30.44
